# Effects of Different Drying Methods on the Quality, Nutritional Components, and In Vitro Bioaccessibility of Kiwifruit

**DOI:** 10.1002/fsn3.71103

**Published:** 2025-10-16

**Authors:** Mengdi Hao, Zhaoqing Yu, Liying Niu, Linlin Han, Mingfei Gu, Yayuan Xu, Zhongyuan Zhang, Cunshan Zhou, Dajing Li, Zhuqing Dai

**Affiliations:** ^1^ School of Food and Biological Engineering Jiangsu University Zhenjiang China; ^2^ Institute of Agro‐Product Processing Jiangsu Academy of Agricultural Sciences Nanjing China

**Keywords:** ascorbic acid, bioaccessibility, carotenoids, drying method, kiwifruit, polyphenols

## Abstract

This study systematically compared the effects of vacuum freeze drying (FD), vacuum microwave drying (MVD), hot air drying (HAD), and their combined drying methods (MVD‐FD, HAD‐FD, MVD‐HAD) on the physical properties, nutritional components, and bioaccessibility of active compounds in kiwifruit. Two typical kiwifruit varieties, Zespri Green and Zespri SunGold, were used as raw materials. The results indicated that both FD and MVD‐FD were effective in preserving kiwifruit color, while the MVD‐FD samples additionally possessed moderate hardness and favorable textural properties. The FD samples displayed a uniform porous structure, while the MVD‐FD samples showed variably compressed pores. In terms of nutrient retention, the FD and MVD‐FD methods yielded the highest contents of total acid, total sugar, polyphenols, ascorbic acid, lutein, and zeaxanthin. In vitro digestion demonstrated that MVD‐FD significantly enhanced the bioaccessibility of active compounds compared to FD, including polyphenols, ascorbic acid, lutein, and zeaxanthin. In conclusion, MVD‐FD is considered a suitable method for producing high‐quality kiwifruit dried products with enhanced nutritional value.

## Graphical abstract

FD‐ and MVD‐FD‐treated kiwifruit slices showed optimal quality and the highest nutrient retention, while MVD‐FD further enhanced nutrient bioaccessibility. Overall, MVD‐FD was identified as the optimal drying method.

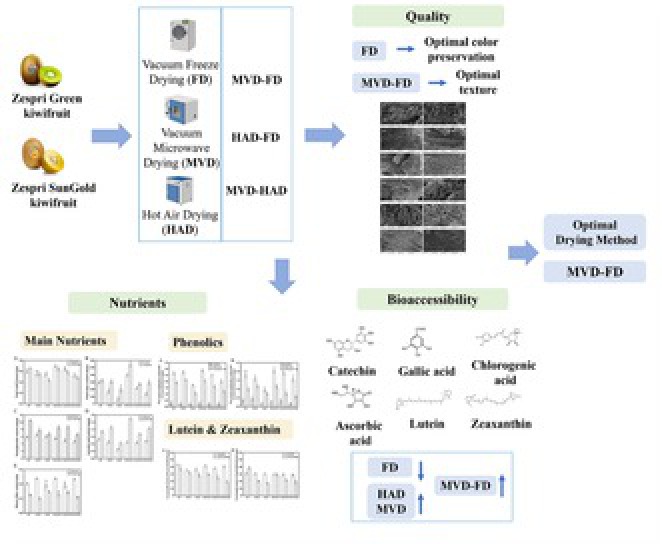

## 1 Introduction

The *Actinidia deliciosa* , known as the kiwifruit, originates from the hilly areas of southern China and boasts considerable nutritional benefits (Zhao et al. 2022). Kiwifruit is rich in diverse bioactive substances, including organic acids, vitamins, polyphenols, carotenoids, minerals, and dietary fiber (Dias et al. 2020). As one of the most popular fruits worldwide, it possesses significant potential health benefits (Yildiz 2022). It has been reported to exhibit various physiological functions, including anti‐tumor, antioxidant, and intestinal health maintenance properties (Richardson et al. 2018).

Kiwifruit is prone to physicochemical degradation and microbial spoilage due to its high moisture content (~80%) (Vincenzo et al. 2024; Yildiz 2022), leading to a short shelf life and thus often causing seasonal supply–demand imbalances. Therefore, reducing water activity through drying processes to extend shelf life has become the primary processing method for kiwifruit. Studies have shown that different drying methods significantly impact the microstructure, nutritional content, bioaccessibility, color, and other attributes of food products (Belwal et al. 2022). Commonly used drying methods include hot air drying (HAD), vacuum microwave drying (MVD), and vacuum freeze drying (FD). Hot air drying, with its simple principle, is applicable to a wide range of materials. However, its long drying cycle and high temperatures often lead to the degradation of key nutrients, flavors, and colors (An et al. 2016). FD is a process that removes moisture from frozen materials through sublimation under vacuum conditions (Salehi 2023). This method is highly effective in preserving the quality and nutritional value of the materials (Wang et al. 2023). Nevertheless, its extended drying time and high energy consumption limit its widespread application (Li et al. 2020). As an emerging drying method, MVD combines vacuum and microwave effects to accelerate the drying process (González‐Cavieres et al. 2021). Due to its low drying temperature and short processing time, it effectively minimizes the loss of heat‐sensitive nutrients (Li et al. 2020). Zhang et al. (2024) found that compared to freeze drying, MVD of kiwifruit pomace offered shorter processing time, lower energy consumption, and comparable product quality. Nazmi Izli et al. (2017) reported that microwave drying required the least time for kiwifruit, while freeze drying was more conducive to retaining polyphenol content and antioxidant capacity. Research by Li et al. (2020) demonstrated that the MVD‐FD significantly reduced drying time and achieved the best results in maintaining quality and reducing hygroscopicity of citrus fruits. Jiang et al. (2017) showed that compared to FD, FD‐MVD for okra samples not only improved product quality but also reduced drying time and energy consumption by approximately 75.36% and 71.92%, respectively. These findings indicate that combined drying methods hold significant application potential as an efficient and high‐quality drying method. Optimizing combined drying processes represents an important direction for future development in drying technology.

Food nutrients, particularly heat‐sensitive compounds like polyphenols and carotenoids, are at risk of degradation during drying processes. The study revealed that HAD, MVD, and other dehydration methods significantly decreased polyphenol and carotenoid levels in fruits and vegetables, consequently impairing their antioxidant, anti‐diabetic, and other biological activities (Ali et al. 2020; Chuyen et al. 2017; Naidu et al. 2016). Simultaneously, the change in food matrix structure during drying affects the release and bioaccessibility of bioactive substances. For instance, the cellular barriers in carrots and bell peppers break down under HAD, which enhances carotenoid liberation during digestion (Zhang et al. 2018). Similarly, freeze drying, vacuum drying, hot air drying, and infrared drying can promote the bioaccessibility of polyphenols in persimmons (Kayacan et al. 2020). However, the effects of different drying methods on the bioaccessibility of key bioactive compounds in kiwifruit remain unexplored. The variation patterns and release characteristics of nutrients during kiwifruit processing require further investigation.

Based on this, the present study employs Zespri Green and SunGold kiwifruits as raw materials to analyze and compare how various drying techniques influence key nutritional components, including total acids, vitamins, polyphenols, and carotenoids. It further investigates how structural changes in the kiwifruit matrix regulate nutrient bioaccessibility, with the goal of offering valuable insights to enhance kiwifruit processing methods and industry practices.

## 2 Materials and Methods

### 2.1 Material Collection

Fresh kiwifruits (Zespri Green and Zespri SunGold) were acquired from Xiaolingwei Farmers Market (Nanjing, China). Folin–Ciocalteu reagent (BR) was supplied by Macklin Biochemical Technology Co. Ltd. (Shanghai, China). 2,6‐Dichloroindophenol (98%) and Coomassie Brilliant Blue G‐250 (AR) were sourced from Lanji Technology Development Co. Ltd. (Shanghai, China). Hydrochloric acid was procured from Kelong Chemical Reagent Factory (Chengdu, China). β‐Carotene (96%), gallic acid (99%), ascorbic acid (AR), and rutin (HPLC ≥ 98%) were supplied by Yuanye Bio‐Technology Co. Ltd. (Shanghai, China). Ethanol, sodium bicarbonate, sodium nitrite, aluminum nitrate, sodium acetate, n‐hexane, and methanol (all domestic analytical grade) were sourced from Sinopharm Chemical Reagent Co. Ltd. (Beijing, China).

### 2.2 Different Drying Methods for Kiwifruit

Following a thorough wash and peel, the fresh kiwifruit samples were sliced into 7‐mm‐thick pieces, rinsed with distilled water, and processed using different drying methods until reaching the target moisture content of 4% ± 0.5% (wet basis). Detailed parameters for each drying method are provided in Table 1. In combined drying methods, MVD‐FD refers to vacuum microwave drying followed by vacuum freeze drying. HAD‐FD involved hot air drying to approximately 60% moisture content, followed by vacuum freeze drying. MVD‐HAD comprised vacuum microwave drying followed by hot air drying.

**TABLE 1:** Six different drying methods and conditions.

Drying method	Drying system	Drying parameters	Drying time
FD	Vacuum freeze dryer (BLK‐FD‐0.5, Jiangsu Bleeker Freezing Technology Development Co. Ltd., Changzhou, Jiangsu)	Cold trap temperature −40°C, vacuum pressure below 50 Pa	18 h
MVD	Vibratory microwave vacuum drying equipment (XMJD6SW‐2, Nanjing Xiaoma Electromechanical Equipment Factory, Nanjing, China)	Microwave power 5 W/g, chamber pressure 0.08 MPa, microwave irradiation for 2 min per cycle with an equal on/off ratio of 1:1	10 min
HAD	Electric heating constant temperature blast drying oven (DHG‐907385‐III, Xinmiao Medical Instrument Manufacturing Co. Ltd., Shanghai, China)	Drying temperature 60°C	10 h
MVD‐FD		MVD: power 5 W/g, FD: cold trap temperature −40°C, vacuum pressure below 50 Pa	MVD: 2 min FD: 18 h
HAD‐FD		HAD: drying temperature 60°C, FD: cold trap temperature −40°C, vacuum pressure below 50 Pa	HAD: 3 h FD: 18 h
MVD‐HAD		MVD: power 5 W/g HAD: drying temperature 60°C	MVD: 2 min HAD: 6 h

### 2.3 Determination of Moisture Content

The moisture content was measured using the method described by Nyangena et al. (2019). Five grams of the fresh sample were dried at 105°C until reaching a stable weight. The moisture content was calculated using the following equation based on the observed mass difference:

$$\%\text{Moisture}=\frac{M_{1}-M_{2}}{M_{1}}\times 100$$

where *M*_1_ is defined as the initial sample mass and *M*_2_ represents the final mass after drying.

### 2.4 Color Analysis

The CM‐700d1 colorimeter (Konica Minolta, Japan) was used for the determination. The colorimeter was zero‐corrected and whiteboard‐corrected to measure the color of different kiwifruit slices. L* denotes the lightness index on a scale from 0 (pure black) to 100 (pure white). Meanwhile, the a* axis indicates the red‐green spectrum (with positive values leaning toward red and negative values leaning toward green), while the b* axis tracks the yellow‐blue range (where positive values shift toward yellow and negative values shift toward blue) (Bhat et al. 2022). The total color difference ΔE is calculated using the following formula:

$$∆E=\sqrt{{\left(L^{*}-L_{0}\right)}^{2}+{\left(a^{*}-a_{0}\right)}^{2}+{\left(b^{*}-b_{0}\right)}^{2}}$$

*L*_0_, *a*_0_, *b*_0_ represent the measurement readings for fresh kiwifruit slices; L*, a*, b* denote the measurements of kiwifruit slices after different drying methods.

### 2.5 Texture Determination

The textural properties of the samples, including hardness, elasticity, and viscosity, were quantitatively analyzed using a CT3 texture analyzer (CNS Farnell, UK). The test conditions were as follows: probe model TA4/1000 cylindrical probe, TA‐BT‐KIT fixture. The operation mode was compression‐type testing. The target value was set at 7.0 mm, with a test distance of 3 mm, a testing speed of 0.5 mm/s, and a return speed of 0.50 mm/s post‐measurement. The maximum force peak (g) in the coordinate graph represents the hardness value. Viscosity is expressed as the negative area S3 between the first compression curve's zero point and the second curve's onset in the coordinate chart in g. Elasticity is characterized by the ratio of the recovery height (T2) after the second compression to the initial compression deformation (T1) in the coordinate chart (Zhang et al. 2018).

### 2.6 Determination of Total Acid

Total acid content was measured by titrating 4 g samples with 0.1 M NaOH to an endpoint pH of 8.5. The consumption of NaOH was recorded, with the total acid content determined via the lactic acid conversion coefficient (mg/mL) (Gao et al. 2022).

### 2.7 Determination of Total Sugar

The dried kiwifruit samples were ground by a tissue grinder, and an appropriate amount of powder was weighed in deionized water. Following ultrasonic‐assisted extraction (240 W, 30 min), the suspension was filtered and subjected to a second extraction. Total sugar content in the combined extracts was determined using the phenol‐sulfuric acid method (Chen and Huang 2019). The extract was diluted 20 times, and 1 mL of sample solution was transferred to a test tube. Next, 2 mL of ethanol and 1 mL of a 5% phenol solution were added stepwise, with thorough mixing after each addition. The mixture was then treated with 5 mL of concentrated sulfuric acid while being vigorously shaken. Following 20 min incubation, absorbance was recorded at 490 nm against a blank reference.

### 2.8 Determination of Phenolic Compounds

Dried kiwifruit slices were ground into powder using a fully automatic sample rapid grinder (Tissuelyser‐48, Shanghai Jingxin Industrial Development Co. Ltd., China). The appropriate amount of powder was weighed in 50% (v/v) ethanol solution, and ultrasonic (240 W) assisted extraction was performed for 30 min. Following centrifugation at 8000 rpm for 15 min, the residue underwent a second extraction cycle. The combined supernatants were diluted to a final volume of 50 mL. Phenolic compounds were subsequently quantified through the Folin–Ciocalteu colorimetric method (Abozed et al. 2014).

Following filtration through a 0.45 μm organic membrane, the extract was analyzed via High Performance Liquid Chromatography (HPLC) analysis to determine the major phenolic compounds (catechin, gallic acid, and chlorogenic acid). The analysis was performed using an Inertsil ODS column (4.6 mm × 250 mm, 3.5 μm) maintained at 25°C. Detection was performed with a diode array detector at 280 nm, with a mobile phase consisting of 1% acetic acid in water (A) and 1% acetic acid in methanol (B). The gradient elution program was as follows: 10% B at 0 min, 26% B at 10 min, 40% B at 25 min, 65% B at 45 min, 95% B at 55 min, returning to 10% B at 58 min and held until 65 min. The mobile phase flowed at 0.6 mL/min with 20 μL injections.

### 2.9 Determination of Ascorbic Acid

Ascorbic acid concentration was measured according to the titration method described by Bhat et al. (2021).

### 2.10 Determination of Dietary Fiber

The total dietary fiber content was quantified through enzymatic and gravimetric methods in accordance with AOAC 985.29 (AOAC 1995) on dried and ground samples.

### 2.11 Determination of Carotenoid Compounds

The dried kiwifruit slices were pulverized with a fully automatic sample rapid grinder. An appropriate amount of powder was weighed and mixed with an extraction solvent consisting of hexane: ethanol: acetone (2:1:1, v/v/v). The mixture underwent static extraction at 37°C for 16 h, followed by nitrogen evaporation concentration. The residue was reconstituted in hexane, filtered through a 0.45 μm organic membrane, and subsequently analyzed for carotenoid content via HPLC (Song et al. 2016). The separation was conducted on a YMC‐C_30_ column (4.6 mm × 250 mm, 5 μm) at 25°C, with detection at 450 nm using a diode array detector. The mobile phase consisted of water: methyl tert‐butyl ether (MTBE): methanol (5:20:75, v/v/v) (A) and water: MTBE: methanol (5:85:10, v/v/v) (B), with the following gradient program: 95% A at 0 min, 80% A at 4.5 min, 50% A at 12.5 min, 25% A at 18 min, 5% A at 24 min, returning to 95% A at 30 min. The flow rate was maintained at 0.6 mL/min with an injection volume of 20 μL.

### 2.12 Scanning Electron Microscopy (SEM)

The dried kiwifruits were cut into 1 cm cubes and mounted on specimen stubs using conductive adhesive. Following gold sputter coating of the cross‐sections, microstructural examination was performed using an S‐4800 field emission scanning electron microscope (EVO‐LS10, Zeiss, Germany) operated at 5 kV accelerating voltage with 100× magnification.

### 2.13 In Vitro Digestion

In vitro simulated digestion assay was performed according to Brodkorb et al. (2019) and Minekus et al. (2014). The dried kiwifruit samples were ground into powder (60 Hz, 45 s) prior to digestion. Subsequently, 3 mL of simulated saliva (pH 6.8) was added to the sample and homogenized for 30 s, followed by the addition of 10 mL of simulated gastric fluid containing pepsin (21 g/L). The pH was adjusted to 2.0 using 1 M HCl, and the mixture was incubated at 37°C with shaking (120 r/min) for 1 h under a nitrogen atmosphere. Then, 2 mL of simulated intestinal fluid containing pancreatin and bile salts was added. The pH was adjusted to 6.9 with 1 M NaHCO_3_ solution, and incubation continued for another 2 h under the same conditions.

After digestion was completed, the digested solution was centrifuged at 4°C, 10,000× *g* for 60 min. The upper micelles were taken for carotenoid extraction assay according to 2.11, and the supernatant was taken for phenolic compounds and ascorbic acid extraction assay according to 2.8 and 2.9. Bioaccessibility is the ratio of the content of the active substance detected by extraction after digestion to the content of the substance in kiwifruit (Tomas et al. 2017).

### 2.14 Statistical Analysis

All experiments were repeated three times, and the data are expressed as mean values with corresponding standard deviations (MEAN ± SD). The Shapiro–Wilk test and Levene's test were used to verify data normality and homogeneity of variance. One‐way analysis of variance (ANOVA) and Tukey's HSD test with a 95% confidence level (*p* < 0.05) were performed to evaluate the significant difference. All statistical analyses were performed using Microsoft Excel 2010 and SPSS 22.0 software. Data visualization was conducted using Origin 2024 software.

## 3 Results and Discussion

### 3.1 Effects of Different Drying Methods on the Color of Kiwifruit

Color characteristics serve as an important quality factor that significantly influences the commercial value of dehydrated products. Table 2 reveals that FD and MVD‐FD significantly enhanced the brightness (L*) of Green and SunGold kiwifruits relative to fresh samples (*p* < 0.05). These results significantly exceeded those of the other four drying methods, aligning with the findings of Gao et al. (2024). The value of L* exhibits significant dependence on drying conditions, particularly processing time and temperature (Muhammad et al. 2021). The sublimation process under low temperature and pressure minimizes enzymatic oxidation and pigment degradation, effectively preventing browning (Zhao et al. 2025). In contrast, both HAD and MVD processes, characterized by elevated temperatures and prolonged drying durations, accelerate chlorophyll degradation, pigment breakdown, and the Maillard reaction (Ucar and Karadag 2019). The combination of MVD and FD demonstrates optimized performance by accelerating the drying rate while maintaining browning inhibition through reduced thermal exposure time (Gao et al. 2024).

**TABLE 2:** Effects of different drying methods on the color of kiwifruits.

Method		L*	a*	b*	△E
Green kiwifruit	Fresh	30.40 ± 1.86	−1.88 ± 0.20	5.59 ± 0.73	
	FD	67.53 ± 8.03^a^	−3.10 ± 0.29^d^	17.25 ± 1.90^cd^	39.12 ± 7.12^b^
	MVD	39.95 ± 4.68^b^	0.89 ± 0.51^ab^	17.80 ± 1.21^bc^	16.09 ± 3.21^c^
	HAD	39.75 ± 5.69^b^	1.70 ± 1.29^a^	21.93 ± 2.69^ab^	19.94 ± 1.83^c^
	MVD‐FD	77.33 ± 7.76^a^	−0.03 ± 1.06^bc^	23.16 ± 3.93^a^	50.19 ± 8.40^a^
	HAD‐FD	33.54 ± 10.93^b^	−1.13 ± 1.00^c^	13.48 ± 1.69^d^	12.86 ± 2.77^c^
	MVD‐HAD	36.04 ± 5.57^b^	0.82 ± 0.31^ab^	17.03 ± 3.74^cd^	13.70 ± 4.80^c^
SunGold kiwifruit	Fresh	31.99 ± 1.66	−0.26 ± 0.39	4.37 ± 0.28	
	FD	71.71 ± 7.37^a^	−0.05 ± 0.31^b^	17.57 ± 3.99^bc^	42.12 ± 6.56^a^
	MVD	29.07 ± 10.37^c^	2.75 ± 2.36^a^	14.89 ± 4.10^c^	14.23 ± 6.11^c^
	HAD	51.45 ± 10.46^b^	1.47 ± 1.36^ab^	21.96 ± 7.86^ab^	26.58 ± 12.40^b^
	MVD‐FD	72.48 ± 7.53^a^	0.02 ± 0.48^b^	18.00 ± 2.12^bc^	42.77 ± 7.52^a^
	HAD‐FD	52.83 ± 5.19^b^	1.09 ± 0.34^ab^	25.22 ± 1.06^a^	29.67 ± 4.05^b^
	MVD‐HAD	44.55 ± 3.93^b^	1.53 ± 0.56^ab^	20.42 ± 3.36^abc^	20.61 ± 4.39^bc^

*Note:* Different lowercase letters in the same column represent significant differences (*p* < 0.05).

The Green and SunGold kiwifruits processed by FD, MVD‐FD, and HAD‐FD all exhibited lower a* values, demonstrating better retention of green coloration. FD‐treated Green kiwifruit showed notably reduced a* values when contrasted with other drying methods (*p* < 0.05). Similarly, Yildiz et al. (2025) observed the lowest a* values in freeze‐dried sweet potatoes, suggesting that FD can effectively preserve the original color of kiwifruit. Conversely, samples treated with MVD, HAD, and MVD‐HAD showed higher a* values, indicating that the color was red. The study by Fakhreddin Salehi et al. (2024) also indicated that the a* value of eggplant slices subjected to HAD after microwave pretreatment was significantly higher than that of untreated samples. This phenomenon may be attributed to prolonged exposure to hot air, moisture, and enzymes, leading to enzymatic browning caused by oxidation and high temperatures. Additionally, non‐enzymatic reactions and heat‐induced pigment degradation further contributed to the observed color changes (Gao et al. 2024). The b* values of kiwifruit were significantly increased after six drying methods, resulting in a more yellowish hue in the dried slices.

The ΔE values of FD and MVD‐FD processed kiwifruits significantly exceeded those of other methods (*p* < 0.05). Ozay‐Arancioglu et al. (2022) used FD, HAD, vacuum drying, and ultrasonic‐assisted vacuum drying methods to dry pomegranate aril samples. Among them, the ΔE obtained by the FD method was the largest, which may be due to the significant increase in L* and the low a* value. Overall, these results indicate that FD and MVD‐FD were effective in enhancing the brightness of kiwifruit products, whereas MVD and HAD caused darkening and a red color shift.

### 3.2 Effects of Different Drying Methods on the Texture of Kiwifruit

Hardness, among the textural parameters, serves as the primary textural parameter for assessing the organoleptic quality of the product. Sensory scores demonstrated a significant inverse correlation with hardness (Chen et al. 2012). Table 3 shows similar drying method impacts on texture for Green and SunGold kiwifruits. The FD samples exhibited significantly reduced hardness (*p* < 0.05), while HAD samples showed notably higher values (*p* < 0.05). The hardness of kiwifruit after MVD was in the middle of the range of FD and HAD. Comparable results were reported by Wang et al. (2021) and Yildiz (2022). Among the combined drying methods, kiwifruits treated with MVD‐FD exhibited the lowest hardness, while those subjected to MVD‐HAD showed the highest hardness. Notably, the MVD‐FD samples maintained moderate hardness coupled with optimal viscoelastic properties. This may be because during freeze‐drying, the water in the dried fruit is frozen into ice crystals, which sublimate and leave holes, making the material more loosely organized (Qiu et al. 2022). The HAD causes slow water evaporation, and kiwifruit slices shrink significantly in volume due to high temperatures (Cai et al. 2017), resulting in the maximum hardness.

**TABLE 3:** Effects of different drying methods on the texture of kiwifruits.

Method		Hardness (g)	Viscosity (g)	Elasticity
Green kiwifruit	FD	3837.67 ± 78.26^e^	314.67 ± 5.03^a^	0.11 ± 0.01^b^
	MVD	6556.67 ± 78.04^c^	52.00 ± 2.00^c^	0.08 ± 0.02^c^
	HAD	9250.67 ± 193.42^a^	3.83 ± 0.76^e^	0.14 ± 0.01^a^
	MVD‐FD	5392.33 ± 209.38^d^	254.00 ± 8.00^b^	0.07 ± 0.01^c^
	HAD‐FD	7296.33 ± 1176.26^bc^	36.00 ± 1.00^d^	0.07 ± 0.01^c^
	MVD‐HAD	8118.67 ± 118.24^b^	37.67 ± 1.52^d^	0.09 ± 0.02^bc^
SunGold kiwifruit	FD	3989.00 ± 517.97^f^	539.67 ± 13.05^a^	0.04 ± 0.01^a^
	MVD	11456.33 ± 1116.33^c^	316.67 ± 56.05^cd^	0.04 ± 0.00^a^
	HAD	16649.67 ± 677.46^a^	260.33 ± 21.46^e^	0.05 ± 0.00^a^
	MVD‐FD	6640.33 ± 356.90^e^	437.33 ± 22.74^b^	0.04 ± 0.01^a^
	HAD‐FD	8136.00 ± 115.33^d^	263.67 ± 9.07^de^	0.04 ± 0.00^a^
	MVD‐HAD	14612.67 ± 986.98^b^	324.00 ± 10.82^c^	0.04 ± 0.00^a^

*Note:* Different lowercase letters in the same column represent significant differences (*p* < 0.05).

### 3.3 Effects of Different Drying Methods on Nutritional Components of Kiwifruit

**FIGURE 1:**
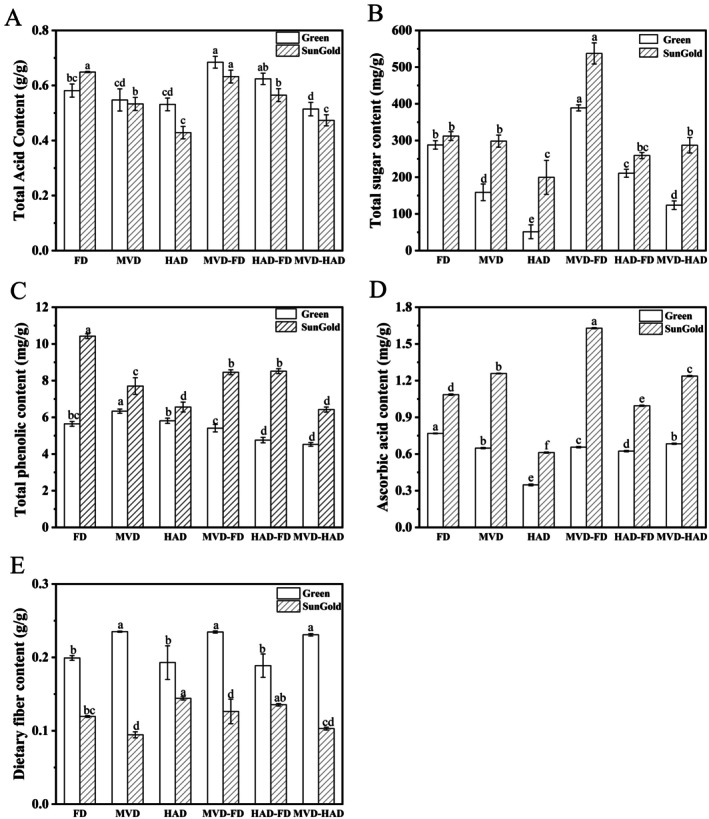
Effects of different drying methods on the main nutrients in kiwifruits. (A) Total acid content. (B) Total sugar content. (C) Total phenolic content. (D) Ascorbic acid content. (E) Dietary fiber content. Different letters above the columns indicate significant differences (*p* < 0.05).

The changes in the content of total acid, total sugar, total phenol, ascorbic acid, and dietary fiber in kiwifruit after applying different drying methods are presented in Figure 1. In fact, differences in fruit types and processing methods can lead to variations in their nutritional components (Rakariyatham et al. 2025).

The Green kiwifruit exhibited a slightly elevated total acid content relative to the SunGold kiwifruit. Among the Green kiwifruit, MVD‐FD yielded the highest total acid content (0.68 ± 0.02 g/g), while the MVD‐HAD resulted in the lowest (0.51 ± 0.02 g/g). For SunGold kiwifruit, FD and MVD‐FD showed relatively higher total acid contents (0.65 ± 0.00 and 0.63 ± 0.02 g/g, respectively) compared to other methods (*p* < 0.05). Overall, the MVD‐FD‐treated kiwifruits maintained 5.21%–37.36% higher total acid content compared to other drying methods.

The MVD‐FD treated Green and SunGold kiwifruits exhibited significantly higher total sugar contents (388.91 ± 8.34 and 537.26 ± 28.75 mg/g, respectively) compared with other drying methods, showing 25.97%–86.83% and 41.89%–62.84% increases, respectively. The HAD‐treated Green and SunGold kiwifruits had the lowest total sugar content (51.24 ± 18.89 and 199.67 ± 46.36 mg/g, respectively), with the Green kiwifruit showing significantly lower levels than all other methods (*p* < 0.05). This phenomenon may result from oxidative degradation of kiwifruit slices during prolonged oxygen‐rich drying, where sugar molecules experience the oxidation of hydroxyl groups and the disruption of hydrogen bonds between molecules (Shang et al. 2021). These chemical processes ultimately decrease the total sugar content. Zhang et al. (2023) demonstrated that freeze‐dried blueberry pomace exhibited the highest total sugar content, followed by MVD, while HAD resulted in a significant reduction in total sugar content. Elevated temperatures and prolonged drying time induced browning reactions in both fructose and glucose, thereby decreasing total sugar levels (Wall and Gentry 2007). The vacuum and low‐temperature conditions of freeze drying and MVD mitigated sugar browning reactions, leading to better preservation of sugar content (Zhang et al. 2023).

The phenolic content was higher in SunGold kiwifruit than in Green kiwifruit. Among all methods, FD‐treated SunGold kiwifruit demonstrated the highest content of total phenolics (10.42 ± 0.13 mg/g), which was higher by 18.88%–38.37% compared to the other methods. This was followed by HAD‐FD and MVD‐FD. The higher retention of phenolic compounds in FD samples was also reported from other studies (Domin et al. 2020; Ozkan et al. 2022; Turkmen et al. 2020). This phenomenon is explained by the synergistic action of vacuum processing and reduced drying temperatures (Chen et al. 2020). In addition to aiding in the preservation of polyphenols, the low temperatures employed in freeze drying can also effectively inhibit polyphenol oxidase activity (Kayacan et al. 2020). The total phenolic content of SunGold kiwifruit was the lowest in HAD and MVD‐HAD, which were 6.56 ± 0.27 and 6.42 ± 0.16 mg/g, respectively, and significantly lower than the other methods (*p* < 0.05). The Green kiwifruit subjected to MVD‐HAD exhibited the lowest total phenol content at 4.52 ± 0.10 mg/g. According to Bhat et al. (2022), the loss of polyphenols during the drying process might result from both polyphenol oxidase activity and elevated temperatures. Ozay‐Arancioglu et al. (2022) reported that freeze drying and HAD yielded the highest and lowest total phenolic content in pomegranate arils, respectively. Wang et al. (2023) found that compared to MVD, freeze drying resulted in the highest total phenolic content in *Cardamine violifolia*, while both HAD and MVD led to a reduction in total phenolic content. This indicates that temperature and vacuum level play critical roles in the retention and stability of total polyphenols. HAD transfers heat from the exterior to the interior of the food material (Yildiz 2022). During this process, moisture is initially removed from the surface, causing cellular hardening and microstructural damage, which subsequently affects the release of bound phenolic compounds (Kayacan et al. 2020). Phenolic compounds are known to be sensitive to both thermal treatments and oxidative processes. The extended thermal exposure during HAD leads to significant degradation of phenolic compounds (Kayacan et al. 2020). Research has indicated that higher microwave power reduces phenolic levels in MVD samples (Dash et al. 2023). The Green kiwifruit treated with MVD exhibited the highest total phenolic content at 6.34 ± 0.12 mg/g, which may be attributed to the low‐temperature vacuum environment and rapid microwave heating that minimized phenolic loss (Dash et al. 2023). Additionally, microwave drying utilizes radiation and penetrating power to disrupt the covalent bonds of intracellular polymers, facilitating the release and collection of substances such as polyphenols (Xu et al. 2022).

As a water‐soluble essential nutrient, ascorbic acid is highly unstable due to its sensitivity to light, temperature, and oxygen, resulting in rapid degradation (Zia and Alibas 2021). SunGold kiwifruit contained abundant ascorbic acid. The highest content (1.63 ± 0.01 mg/g) was observed after MVD‐FD, representing a 22.75%–62.44% increase compared to other methods. In Green kiwifruit, the FD method yielded the highest ascorbic acid content (0.77 ± 0.01 mg/g). In contrast, HAD resulted in the lowest levels of ascorbic acid among all methods for both cultivars, with contents of 0.61 ± 0.00 mg/g in SunGold kiwifruit and 0.34 ± 0.00 mg/g in Green kiwifruit (*p* < 0.05). Research by Yildiz et al. (2025) indicates that sweet potato samples processed via HAD exhibited the lowest ascorbic acid content, while those treated with FD showed the highest levels. Gao et al. (2024) found that ascorbic acid content in dried white apricot products produced by freeze‐drying and freeze‐drying combined with MVD was significantly higher than in MVD and HAD‐treated samples. This significant difference mainly stems from the continuous drying in both MVD and HAD. Research by Gamboa‐Santos et al. (2014) indicates that ascorbic acid loss is primarily attributable to two key factors: drying time and temperature. Extended thermal contact during the drying stage causes enzymatic breakdown and heat‐induced degradation of ascorbic acid, leading to a significant reduction in its content (Wang et al. 2018). MVD retains more ascorbic acid than HAD because of its shorter drying time and reduced exposure to hot air, leading to less nutrient degradation. Consequently, the low‐temperature, low‐oxygen environment of FD and MVD‐FD proves more conducive to preserving ascorbic acid (Gao et al. 2024).

The dietary fiber content of kiwifruit changed after different drying methods. The dietary fiber content of Green kiwifruit after MVD, MVD‐FD, and MVD‐HAD was 0.24 ± 0.01, 0.23 ± 0.00, and 0.23 ± 0.01 g/g, respectively, which was significantly higher than the dietary fiber content after FD, HAD, and HAD‐FD (0.20 ± 0.01, 0.19 ± 0.02, 0.19 ± 0.02 g/g, *p* < 0.05). The dietary fiber content of SunGold kiwifruit after different drying methods ranged from 0.09–0.14 g/g, with the highest dietary fiber content of 0.14 ± 0.00 g/g after HAD and the lowest content of 0.09 ± 0.00 g/g after MVD.

**FIGURE 2:**
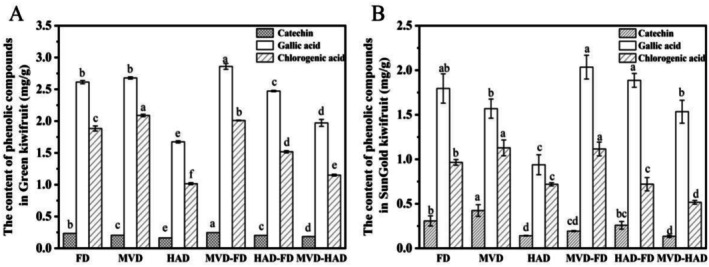
Effects of different drying methods on the content of catechin, gallic acid, and chlorogenic acid in kiwifruits. (A) The content of phenolic compounds in Green kiwifruit. (B) Content of phenolic compounds in SunGold kiwifruit. Different letters above the columns indicate significant differences (*p* < 0.05).

Changes in three characteristic phenolic compounds—catechin, gallic acid, and chlorogenic acid—under different drying methods are presented in Figure 2. The catechin content of Green kiwifruit treated with MVD‐FD and FD was significantly higher than that of the other methods, whereas the catechin content of SunGold kiwifruit treated with MVD and FD was significantly higher than that of the other methods, and the lowest catechin content was found in the HAD method. The data reveal that the trends of gallic acid and chlorogenic acid were similar in the drying methods of Green and SunGold kiwifruits. The gallic acid contents of Green and SunGold kiwifruits after MVD‐FD were 2.86 ± 0.05 and 2.04 ± 0.31 mg/g, respectively, and significantly higher than the other methods (*p* < 0.05). The content of gallic acid in Green and SunGold kiwifruits treated with HAD was 1.67 ± 0.02 and 0.94 ± 0.11 mg/g, respectively, which was significantly lower than that in the other methods (*p* < 0.05). The chlorogenic acid contents of Green kiwifruit after drying by MVD and MVD‐FD were 2.09 ± 0.02, 2.01 ± 0.01 mg/g, and those of SunGold kiwifruit were 1.13 ± 0.09 and 1.12 ± 0.08 mg/g, which were significantly higher than the other methods (*p* < 0.05).

**FIGURE 3:**
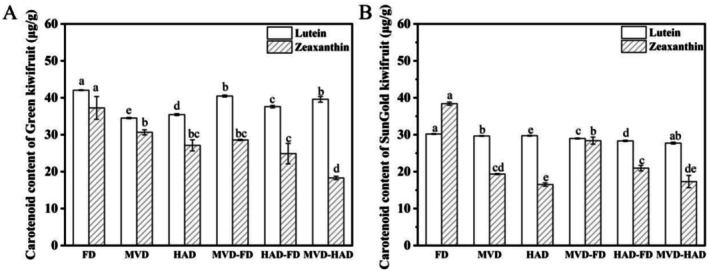
Effects of different drying methods on the content of lutein and zeaxanthin acid in kiwifruits. (A) Carotenoid content of Green kiwifruit. (B) Carotenoid content of SunGold kiwifruit. Different letters above the columns indicate significant differences (*p* < 0.05).

HPLC analysis revealed that lutein and zeaxanthin were the predominant carotenoids in kiwifruit. These isomeric compounds exhibited significant antioxidant properties, including free radical scavenging capacity and anti‐inflammatory activity (Dong et al. 2025). The concentration profiles of these two carotenoids are presented in Figure 3. The effects of different drying methods on the lutein and zeaxanthin contents were similar between Green and SunGold kiwifruits. FD treatment yielded the highest contents of these carotenoids, which were significantly greater (*p* < 0.05) than all other methods. The lutein and zeaxanthin levels in FD‐treated Green kiwifruit were 42.06 ± 0.09 and 37.26 ± 3.08 μg/g, respectively, while those in SunGold kiwifruit were 30.20 ± 0.09 and 38.41 ± 0.43 μg/g. Consistent results were reported by Kayacan et al. (2020). The FD method is characterized by low contact temperature and lower partial pressure of oxygen, which may result in a high retention rate of lutein and zeaxanthin components (Regier et al. 2005). The lutein and zeaxanthin levels after MVD‐FD were the next highest, and the lowest levels were found in the HAD or MVD‐HAD. The study by Li et al. (2020) also demonstrated that freeze‐dried citrus samples exhibited the highest carotenoid content, while those treated with MVD‐FD showed higher levels than those dried by MVD alone. Carotenoids are prone to oxidation due to their high degree of unsaturation, while thermal treatment can lead to their degradation or isomerization (Fratianni et al. 2010).

### 3.4 Effects of Different Drying Methods on the Microstructure of Kiwifruit

**FIGURE 4:**
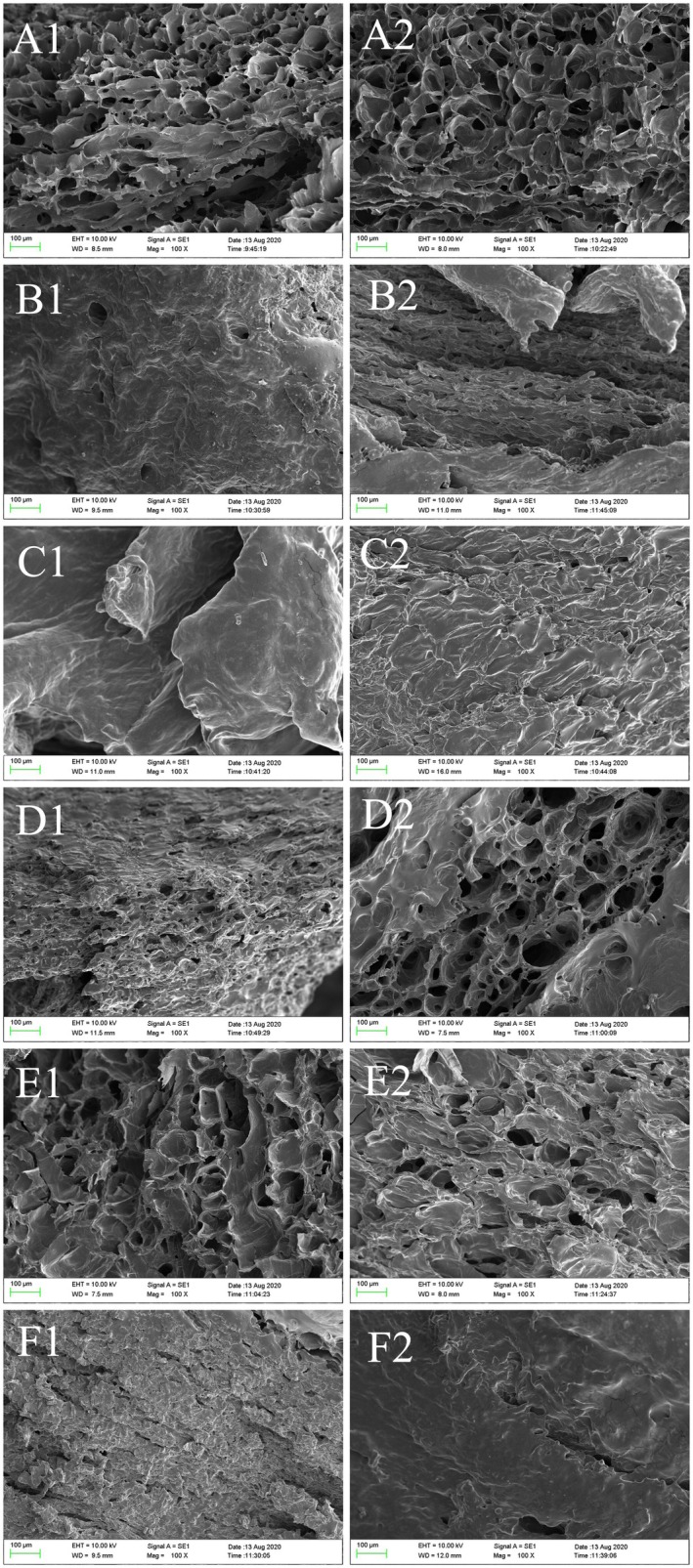
Effects of different drying methods on the microstructure (magnification of 100×) of kiwifruits: (A) FD, (B) MVD, (C) HAD, (D) MVD‐FD, (E) HAD‐FD, and (F) MVD‐HAD. 1 and 2 represent Green and SunGold kiwifruits, respectively.

Scanning electron microscopy (magnification of 100×) images are shown in Figure 4. The kiwifruit after FD showed a loose and porous structure with uniform and well‐arranged pores. This is due to the vaporization of water under vacuum conditions, transitioning directly from solid to vapor without passing through a liquid phase. This effectively prevented the collapse of the sample surface and ensured the structural integrity of the samples (Zhao et al. 2023). The positive effects of FD on the structural and cellular integrity of the samples were also demonstrated in a related study (Varley et al. 2016). Both MVD and HAD methods resulted in increased firmness and hardening of kiwifruit, with disrupted microporous structures and the formation of dense, rough surface morphology. This is similar to the results of Wang et al. (2023), which showed that the longer drying time and higher temperature resulted in structural shrinkage. The kiwifruit slices treated with MVD‐FD and HAD‐FD still showed a loose and porous structure, but the pore sizes varied and were more compact than those treated with FD, and there were different degrees of extrusion in the tissues. Gao et al. (2024) used different drying methods to treat small white apricots, and the results also showed that the samples after MVD‐FD had a loose and porous structure, which might be due to the enhanced swelling capacity of MVD, resulting in greater internal porosity. Co‐drying combines the advantages of different stages, MVD or HAD stage for rapid dehydration, and FD for porous skeleton formation by ice crystal sublimation at low temperature. However, the pre‐drying stage of combined drying may result in the collapse of some pores due to thermal effects and the formation of structures of different sizes. During heat and mass transfer in porous media under microwave irradiation, moisture gradient‐induced stress within the material leads to product expansion, contraction, and structural rupture (Yue et al. 2023). The MVD‐HAD processed kiwifruit slices exhibited a microscopic morphology similar to that of slices treated with MVD or HAD alone, all showing a hard, knotted, and tight surface morphology.

### 3.5 Effects of Different Drying Methods on the Bioaccessibility of Major Nutrients in Kiwifruit

Bioaccessibility reflects the ease of digestion and absorption of a substance into the human body (de Oliveira et al. 2017). In vitro digestion models have been widely employed to investigate the release kinetics and bioaccessibility of bioactive compounds from food matrices through simulation of gastrointestinal conditions (Santos et al. 2023). The bioaccessibility of major nutrients in kiwifruit after different drying methods is shown in Table 4.

**TABLE 4:** Effects of different drying methods on the bioaccessibility of the main nutrients of kiwifruits.

Method		Bioaccessibility (%)					
		Catechin	Gallic acid	Chlorogenic acid	Ascorbic acid	Lutein	Zeaxanthin
Green kiwifruit	FD	19.55 ± 1.95^d^	17.17 ± 0.96^b^	24.58 ± 1.60^c^	39.42 ± 3.58^e^	36.74 ± 0.90^d^	33.68 ± 0.66^c^
	MVD	32.61 ± 4.13^a^	29.33 ± 3.77^a^	41.43 ± 1.39^a^	59.66 ± 0.76^a^	45.98 ± 0.63^a^	61.18 ± 0.70^a^
	HAD	25.29 ± 2.99^bc^	24.54 ± 3.64^ab^	40.95 ± 2.11^a^	45.22 ± 5.08^de^	42.88 ± 2.57^ab^	60.62 ± 2.96^a^
	MVD‐FD	29.53 ± 3.10^ab^	25.53 ± 7.23^a^	35.90 ± 1.74^b^	56.96 ± 1.00^ab^	40.96 ± 1.15^bc^	52.93 ± 1.24^b^
	HAD‐FD	20.69 ± 2.07^cd^	22.10 ± 3.11^ab^	33.50 ± 2.65^b^	53.12 ± 2.89^bc^	38.65 ± 2.49^cd^	51.48 ± 0.97^b^
	MVD‐HAD	27.90 ± 1.40^ab^	24.36 ± 0.25^ab^	36.80 ± 2.44^b^	47.57 ± 2.55^cd^	44.52 ± 1.83^a^	63.69 ± 1.48^a^
SunGold kiwifruit	FD	18.30 ± 1.52^b^	24.57 ± 1.45^b^	26.07 ± 4.23^b^	42.62 ± 8.80^b^	32.49 ± 2.91^c^	31.34 ± 2.12^e^
	MVD	29.11 ± 4.77^a^	34.74 ± 5.93^a^	39.41 ± 3.17^a^	56.48 ± 3.99^a^	43.65 ± 6.17^b^	59.77 ± 3.88^b^
	HAD	21.17 ± 4.62^ab^	31.27 ± 1.99^ab^	33.40 ± 2.90^a^	51.28 ± 5.17^ab^	42.90 ± 4.72^b^	67.89 ± 4.08^a^
	MVD‐FD	23.06 ± 2.75^ab^	30.83 ± 1.18^ab^	35.28 ± 2.86^a^	55.90 ± 4.48^a^	43.38 ± 3.39^b^	40.51 ± 1.79^d^
	HAD‐FD	19.88 ± 0.58^ab^	31.07 ± 4.08^ab^	33.35 ± 2.49^a^	49.12 ± 0.56^ab^	37.74 ± 3.95^bc^	52.13 ± 1.77^c^
	MVD‐HAD	21.37 ± 8.22^ab^	31.10 ± 5.47^ab^	37.66 ± 3.40^a^	52.19 ± 2.82^ab^	53.09 ± 2.61^a^	62.85 ± 6.16^ab^

*Note:* Different lowercase letters in the same column represent significant differences (*p* < 0.05).

In Green kiwifruit processed with the FD method, the bioaccessibility of catechin, gallic acid, and chlorogenic acid was 19.55% ± 1.95%, 17.17% ± 0.96%, and 24.58% ± 1.60%, respectively. These values were significantly lower than those in samples treated with the MVD and MVD‐FD methods (*p* < 0.05). Meanwhile, MVD increased the bioaccessibility of the three phenolics most significantly. For example, in Green kiwifruit, MVD significantly enhanced the bioaccessibility of catechin, gallic acid, and chlorogenic acid by 66.82%, 70.83%, and 68.53%, respectively, compared to those in the FD‐treated group. Similarly, the bioaccessibility of total phenolics was also higher in co‐treatment groups involving MVD than in groups treated with other methods. After HAD treatment, the bioaccessibility of catechin and chlorogenic acid in Green kiwifruit was significantly higher than that in the FD‐treated group (*p* < 0.05). In SunGold kiwifruit, HAD significantly increased the bioaccessibility of chlorogenic acid compared to the FD‐treated group (*p* < 0.05). Compared to FD, HAD‐FD significantly enhanced the bioaccessibility of chlorogenic acid in both Green and SunGold kiwifruits (*p* < 0.05). These results are consistent with the observations of Kayacan et al. (2020). Polyphenols are absorbed through passive diffusion across the epithelial barrier of the small intestine. Heat treatment increased the in vitro bioaccessibility of polyphenols from kiwifruit, likely by modifying the molecular structure of bioactive compounds and their interactions with other components. These changes ultimately influence their digestion and intestinal absorption (Bai et al. 2021).

The effect of different methods on the bioaccessibility of ascorbic acid showed a similar trend to that of polyphenols. The lowest bioaccessibility of ascorbic acid was recorded in the FD method, which was 39.42% ± 3.58% and 42.62% ± 8.80% in Green and SunGold kiwifruits, respectively. The two drying methods, MVD and MVD‐FD, showed the highest bioaccessibility of ascorbic acid, which was 32.53%–51.35% and 31.16%–44.51% higher than that of the FD‐treated group, respectively. The bioaccessibility of ascorbic acid in Green kiwifruit treated with HAD‐FD was significantly higher than that in the FD‐treated group (*p* < 0.05).

Lutein and zeaxanthin bioaccessibility in kiwifruit reached 32.49% ± 2.91%–36.74% ± 0.90% after FD. MVD and HAD resulted in lutein bioaccessibility of 42.88% ± 2.57%–45.98% ± 0.63% and zeaxanthin bioaccessibility of 59.77% ± 3.88%–67.89% ± 4.08%. MVD‐FD and HAD‐FD also improved the bioaccessibility of lutein and zeaxanthin compared with FD, but lower than MVD and HAD alone.

Overall, the bioaccessibility of polyphenols, ascorbic acid, lutein, and zeaxanthin in kiwifruit was lower in the FD method than in the other methods. Conversely, the bioaccessibility in samples treated with MVD or HAD alone was higher than in their counterparts treated with the combined MVD‐FD or HAD‐FD methods, respectively. After HAD, MVD, and HAD‐MVD treatments, the bioaccessibility of kiwifruit polyphenols reached 21%–41%, ascorbic acid bioaccessibility reached 45%–60%, and lutein compounds bioaccessibility reached 43%–68%. Disruption of food microstructure affects the release of a bioactive compound from the food matrix or microstructure (Parada and Aguilera 2007; Veda et al. 2008), which in turn may influence the bioaccessibility of these compounds during subsequent human consumption (Niamnuy et al. 2014). Tydeman et al. (2010) demonstrated that the digestive juices during digestion only slightly swell the cell walls, and the majority of the cells remained intact after digestion, thus preventing the release of substances such as carotenoids. On the other hand, MVD and HAD methods can disrupt the cell wall structure of plant tissues and promote the entry of nutrients such as polyphenols and carotenoids from the food matrix into the digestive fluid to form micelles (Sun et al. 2017; Zhang et al. 2018). A study by Hiranvarachat et al. (2012) demonstrated that HAD could increase the relative bioaccessibility of β‐carotene in carrots by more than twofold compared to fresh samples. This enhancement is attributed to the fact that the structural integrity of fresh plant cells hinders enzymatic access to carotenoids embedded within the matrix, potentially limiting their release and resulting in lower relative bioaccessibility. Similar results have been reported by other scholars, such as polyphenol bioavailability in berries reaching 45.24%–59.15% after hot air drying and microwave drying (Zhao et al. 2017). The bioaccessibility of β‐carotene in carrots was elevated by 47%–73% under different hot air drying conditions (Hiranvarachat et al. 2012). Kayacan et al. (2020) revealed that ultrasound‐assisted vacuum drying, infrared drying, freeze drying, and hot air drying significantly increased the bioaccessibility of phenolics, which was higher than the other groups by 18.43% after hot air drying.

Therefore, the higher bioaccessibility observed in the MVD‐FD compared to the FD in this study can be primarily attributed to the pretreatment effect of microwave drying, which more significantly disrupted the cellular structure of kiwifruit, thereby facilitating the release of nutrients and consequently enhancing their bioaccessibility. As shown in Section 3.4, FD better preserved the structural and cellular integrity of the samples, whereas both MVD and HAD methods damaged the microporous architecture of kiwifruit, which is conducive to the release of nutritional compounds. Increased microstructural damage allows digestive enzymes to penetrate more easily to reach bioactive compounds and enables a more efficient release of nutrients from the food matrix (Niamnuy et al. 2014). It is important to point out that despite the ability of thermal drying methods to disrupt plant cell walls, large molecules such as starch and fiber in different fruit and vegetable raw materials affect the release of small‐molecule actives from the matrix after drying, thus showing differences in bioaccessibility (Zhang et al. 2018).

## 4 Conclusion

Results demonstrate that both the FD and MVD‐FD methods yielded superior overall quality in dried kiwifruit, particularly in terms of color retention, microstructure preservation, and nutrient conservation. Compared to FD, MVD‐FD resulted in a more desirable texture with moderate hardness and significantly enhanced the bioaccessibility of active compounds. Thus, MVD‐FD can be regarded as the optimal drying method for kiwifruit slice processing. Given that sensory attributes are critical in industrial applications and considering the inherent limitations of the in vitro digestion model employed in this study, further research should systematically incorporate sensory evaluations and in vivo experiments to more comprehensively investigate the absorption and utilization efficiency of bioactive compounds derived from kiwifruit subjected to different drying methods.

## Author Contributions

**Mengdi Hao:** conceptualization (lead), data curation (lead), formal analysis (lead), investigation (lead), methodology (lead), software (lead), validation (lead), visualization (lead), writing – original draft (lead), writing – review and editing (lead). **Zhaoqing Yu:** data curation (supporting), investigation (supporting). **Liying Niu:** methodology (supporting). **Linlin Han:** investigation (supporting). **Mingfei Gu:** visualization (supporting). **Yayuan Xu:** conceptualization (supporting). **Zhongyuan Zhang:** formal analysis (supporting). **Cunshan Zhou:** validation (supporting). **Dajing Li:** funding acquisition (supporting), project administration (lead). **Zhuqing Dai:** funding acquisition (lead), resources (lead), supervision (lead), writing – review and editing (lead).

## Conflicts of Interest

The authors declare no conflicts of interest.

## Data Availability

Research data supporting this study is available upon reasonable request from the corresponding author.
